# Blood pressure control and antihypertensive pharmacotherapy patterns in a hypertensive population of Eastern Central Region of Portugal

**DOI:** 10.1186/1472-6963-10-349

**Published:** 2010-12-30

**Authors:** Manuel P Morgado, Sandra A Rolo, Luísa Pereira, Miguel Castelo-Branco

**Affiliations:** 1 Health Sciences Research Centre, University of Beira Interior, Covilhã, Portugal; 2 Hospital Centre of Cova da Beira, E.P.E., Covilhã, Portugal; 3 Mathematics Department of University of Beira Interior, Covilhã, Portugal

## Abstract

**Background:**

Interventions to improve blood pressure control in hypertension have had limited success in clinical practice despite evidence of cardiovascular disease prevention in randomised controlled trials.

The objectives of this study were to evaluate blood pressure control and antihypertensive pharmacotherapy patterns in a population of Eastern Central Region of Portugal, attending a hospital outpatient clinic (ambulatory setting) for routine follow-up.

**Methods:**

Medical data of all patients that attended at least two medical appointments of hypertension/dyslipidemia in a university hospital over a one and a half year period (from January 2008 to June 2009) were retrospectively analysed. Demographic variables, clinical data and blood pressure values of hypertensive patients included in the study, as well as prescribing metrics were examined on a descriptive basis and expressed as the mean ± SD, frequency and percentages. Student's test and Mann-Whitney rank sum test were used to compare continuous variables and χ^2 ^test and Fisher exact probability test were used to test for differences between categorical variables.

**Results:**

In all, 37% of hypertensive patients (n = 76) had their blood pressure controlled according to international guidelines. About 45.5% of patients with a target blood pressure <140/90 mmHg (n = 156) were controlled, whereas in patients with diabetes or chronic kidney disease (n = 49) the corresponding figure was only 10.2% (*P *< 0.001). Among patients initiating hypertension/dyslipidemia consultation within the study period 32.1% had stage 2 hypertension in the first appointment, but this figure decreased to 3.6% in the last consultation (*P *= 0.012). Thiazide-type diuretics were the most prescribed antihypertensive drugs (67%) followed by angiotensin receptor blockers (60%) and beta-blockers (43%). About 95.9% patients with comorbid diabetes were treated with an angiotensin-converting enzyme inhibitor or an angiotensin receptor blocker.

**Conclusions:**

Clinically important blood pressure decreases can be achieved soon after hypertension medical appointment initiation. However, many hypertensive patients prescribed with antihypertensive therapy fail to achieve blood pressure control in clinical practice, this control being worse among patients with diabetes or chronic kidney disease. As pharmacotherapy patterns seem to coincide with international guidelines, further research is needed to identify the causes of poor blood pressure control.

## Background

Hypertension is a major risk factor in the development of cardiovascular disease, with myocardial infarction and stroke being one of the most important health problems in Portugal causing excess morbidity and mortality [1]. It is estimated that over three million Portuguese adults (about 30% of the Portuguese population) suffer from hypertension [2]. In a recently published survey [2], only 11.2% hypertensives had their blood pressure (BP) controlled (<140/90 mmHg). This figure is even lower for the Central Region of Portugal, where only 9.7% of the total number of hypertensives have their BP controlled [2]. Furthermore, of the total number of Portuguese hypertensives who were aware of having hypertension and reported taking their medication regularly, only 28.9% had their BP controlled. Again, this figure was lower in the Central Region of Portugal, where the rate of control was only 26.1%. The definition of controlled hypertension in this Portuguese survey was considered as mean systolic BP <140 mmHg and diastolic BP of <90 mmHg and did not take into account hypertensive patients with diabetes mellitus or chronic kidney disease (CKD). The seventh report of the Joint National Committee on the Prevention, Detection, Evaluation, and Treatment of High Blood Pressure (JNC 7) set a lower BP target (<130/80 mmHg) for hypertensive patients with diabetes or CKD [3].

The Eastern Central Region of Portugal possesses a university teaching hospital at Covilhã, named Cova da Beira Hospital Centre, with an important hypertension/dyslipidemia outpatient clinic, which serves a significant hypertensive population of the District of Castelo Branco. To better understand the unsatisfactory levels of BP control of this hypertensive population and focus efforts on improving them, it would be useful to know the extent to which hypertensive patients with different risk for vascular complications are not satisfactorily controlled.

Accordingly, a retrospective study was conducted to evaluate the level of BP control in hypertensive patients, attending the afore mentioned hypertension/dyslipidemia outpatient hospital clinic for routine follow-up, according to the JNC 7 guidelines (<140/90 mmHg for general hypertensive patients and <130/80 mmHg for hypertensive patients with diabetes or CKD).

## Methods

### Settings

This study was conducted in a hypertension/dyslipidemia clinic in the university teaching hospital of Cova da Beira Hospital Centre, Covilhã, District of Castelo Branco, located in the Eastern Central Region of Portugal. This outpatient clinic is one of the most important clinics of this region of Portugal in the field of hypertension/dyslipidemia and serves a significant hypertensive population of the Covilhã surrounding area, with a population of 35,000 inhabitants. It should be emphasized that due to a decreased supply of primary care practitioners in this Region of Portugal that has led to a shortage in primary care delivery, this outpatient clinic provides follow-up care to hypertensive patients. This is done by a health care team composed of internal medicine physicians and nurses. Virtually all hypertensive patients managed and monitored in this clinic have essential arterial hypertension.

### Study population

The study population consisted of outpatients that attended the hypertension/dyslipidemia clinic in the university teaching hospital. All patients are adults aged 18 or over. They were included if they had an established diagnosis of arterial hypertension (BP measurements in the clinic of ≥140/90 mmHg) and had attended at least two medical appointments over a one and a half year period (from January 2008 to June 2009). Hypertensive patients without diabetes and/or CKD with BP <140/90 mmHg in their last appointment were considered to have their BP controlled. For hypertensive patients with diabetes or CKD, BP control was defined as BP measurements <130/80 mmHg in their last appointment. Study subjects were also analyzed based on whether they had selected "high-risk" conditions or characteristics listed by JNC 7 as "compelling indications" (i.e., cerebrovascular disease, CKD, heart failure, ischemic heart disease, diabetes) or "special situations" (i.e., obesity, hyperlipidemia, metabolic syndrome, advanced age) and by stage of hypertension (i.e., Stage 1, 140-159/90-99 mmHg; Stage 2, ≥160/100) [3]. Compelling indications for antihypertensive drugs are based on benefits from outcome studies or existing clinical guidelines; the compelling indication is managed in parallel with the BP [3]. Patients with both compelling indications and special indications were classified into the "compelling indications" group. The presence of these conditions among study subjects was ascertained based on available diagnostic information, laboratory/exam values, and treatments/medications recorded anytime during the study period. BP was measured in a seated position after a five-minute rest period, using a mercury sphygmomanometer or semi-automatic device, the mean of two consecutive measurements being recorded.

### Data extraction

Data for this study were retrospectively obtained from the hospital electronic medical records (HEMR) database. The HEMR database of Cova da Beira Hospital Centre is comprised of detailed patient-level clinical and administrative information from all patients that utilized, at least once, this hospital. Available information includes patient demographics, medical problems (including the date on which each medical problem was first diagnosed), various measures of physiological status and medications prescribed. Dates for each medical contact are also provided, allowing all data to be analysed in chronological order. This database is authorized by the Portugal Department of Health, the government department responsible for public health issues and it ensures patient data confidentiality. Since subjects cannot be identified and confidentiality was warranted, institutional review board approval for this study was neither needed nor sought.

### Statistical analysis

Demographic variables, clinical data and BP values of hypertensive patients included in the study, as well as prescribing metrics were examined on a descriptive basis and expressed as the mean ± SD, frequency and percentages. Student's test and Mann-Whitney rank sum test were used to compare continuous variables and χ^2 ^test and Fisher exact probability test were used to test for differences between categorical variables. For the comparison between results in the first and in the last appointment in the same hypertensive patients who initiated hypertension consultation within the study period, the Wilcoxon rank sum test for paired data was used. All statistical analyses were done using SPSS for Windows, version 17.0 (SPSS Inc., Chicago, IL) and a *P*-value of less than 0.05 was considered to indicate statistical significance.

## Results

### Patient characteristics

Overall, 273 patients attended medical appointments at the hypertension/dyslipidemia clinic of Cova da Beira Hospital Centre for routine follow-up during the study period. Among these patients, evaluation of 25 patients was still under course and there wasn't an established diagnosis of arterial hypertension and/or dyslipidemia and 10 patients had a diagnosis of dyslipidemia but not arterial hypertension. Of the remaining 238 patients, with an established diagnosis of arterial hypertension, 33 were excluded from our study because they had only attended one medical appointment. Most of these patients (n = 27) are no longer followed by the hypertension/dyslipidemia clinic due to several reasons (e.g., referral to other hospital clinics, residency change to other region served by a different hospital, renouncement to medical appointments, death); the remaining 6 hypertensive patients had been admitted to the hypertension/dyslipidemia clinic very recently (May or June 2009) and their antihypertensive medication was probably not yet fully titrated. It should be noted that the first months of antihypertensive therapy may be a critical period for many patients, especially for those at the highest risk for adverse outcomes and several alterations of the therapeutic regimen are often required to control BP and adverse effects.

Altogether, 205 hypertensive patients satisfied our inclusion criteria and were included in our statistical analyses. The overall mean age of these patients was 60 ± 12 years, 40.5% being male and 59.5% female (Table 1). Among these patients, 156 (76%) had neither diabetes nor CKD and were considered to have a target BP of <140/90 mmHg, whereas the remaining 49 (24%) had either diabetes and/or CKD and were considered to be controlled with a BP of <130/80 mmHg. Mean ages were 58 ± 12 years for patients without diabetes and CKD, and 66 ± 10 years for patients with either or both these pathologies (*P *< 0.001). There were 37% (58/156) males in the first group and 51% (25/49) in the second group (*P *= 0.085). Most patients were long term hypertensives, with 65% of all patients having high BP for over five years. Only 8 patients have been diagnosed hypertensive for the first time within the last twelve months of the study period.

**Table 1:** Characteristics of hypertensive patients included in the study (n = 205)

Characteristics	n	Frequencies (%)
**Age**		
≥18 - <35 years	4	2.0
35 - 64 years	128	62.4
≥65 years	73	35.6

**Gender**		
Male/Female	83/122	40.5/59.5

**Duration of hypertension**		
<1 year	8	3.9
≥1year and <5 years	83	31.2
≥5 years and <10 years	80	41.5
≥10 years	34	23.4

**Comorbid conditions**		
Cerebrovascular disease	16	7.8
Chronic kidney disease	12	5.9
Diabetes	41	20.0
Heart failure	2	1.0
Ischemic heart disease	8	3.9
Dyslipidemia	122	59.5
Metabolic syndrome	4	2.0
Obesity (body mass index ≥30)	50	24.4
Peripheral arterial disease	5	2.4
**Advanced age (≥65 years)**	73	35.6
**None of the above**	35	17.1

**Target BP values**		
**(JCN 7 guidelines)**		
<140/90 mmHg	156	76.1
<130/80 mmHg	49	23.9

Thirty-one percent of the study population had a compelling indication and 52% had a special situation, as defined by JNC 7. The most common compelling indications were diabetes (20%), cerebrovascular disease (8%) and CKD (6%), whereas dyslipidemia (60%), advanced age (36%) and obesity (24%) were the most common special situations (Table 1).

The distribution of age-groups and target BP values in the hypertensive study population are shown in Table 2. It is worth mentioning that all patients in the age-group ≥18 - <35 years had a BP target value of <140/90 mmHg, and 59.2% patients in the higher cardiovascular risk group were ≥65 years.

**Table 2:** Number of patients in each age group/target BP value combination that achieved BP control

	Target BP values	
Age	<140/90 mmHg	<130/80 mmHg
**≥18 - <35 years**	50% (2/4) patients	0 patients

**35 - 64 years**	54% (58/108) patients	10% (2/20) patients

**≥65 years**	25% (11/44) patients	10% (3/29) patients

### Blood pressure control

Overall, the mean systolic BP of the 205 hypertensive patients included in our analysis was 140.1 ± 15.0 mmHg and the mean diastolic BP was 81.5 ± 11.1 mmHg, with 44% (91/205) patients attaining BP <140/90 mmHg (Table 3). Among the remaining 56% (114/205) patients, with a BP equal or higher than 140/90 mmHg, 45% (92/205) patients had stage 1 hypertension and 11% (22/205) had stage 2 hypertension.

**Table 3:** Blood pressure control of hypertensive patients

BP	All patients (n = 205)	Patients with target BP <140/90 mmHg (n = 156)	Patients with target BP <130/80 mmHg (n = 49)	Patients initiating HT appointments within the study period (n = 28) - First appointment	Patients initiating HT appointments within the study period (n = 28) - Last appointment
**Mean ± SD SBP/DBP (mmHg)**	140.1 ± 15.0/81.5 ± 11.1	138.6 ± 14.4/81.9 ± 10.5	144.6 ± 16.2/80.4 ± 12.9	149.4 ± 17.1/87.3 ± 13.2	138.1 ± 11.0/83.7 ± 9.3

**BP <140/90 (mmHg)**	91 (44%)	71 (45.5%)	20 (41%)	6 (21%)	11 (39%)

**BP ≥140/90 (mmHg)**	114 (56%)	85 (54.5%)	29 (59%)	22 (79%)	17 (61%)

**BP controlled (JCN 7 guidelines)**	76 (37%)	71 (45.5%)	5 (10%)	6 (21.4%)	10 (35.7%)

**BP not controlled**					

**120-139/80-89**	15 (7%)	NA	15 (31%)	0	1 (3.6%)

**Stage 1**	92 (45%)	71 (45.5%)	21 (43%)	13 (46.4%)	16 (57.1%)

**Stage 2**	22 (11%)	14 (9%)	8 (16%)	9 (32.1%)	1 (3.6%)

DBP - Diastolic BP; HT - Hypertension; NA - Not applicable; SBP - Systolic BP.

When we consider the target BP values defined by the JCN 7 guidelines, 45.5% (71/156) patients with neither diabetes nor CKD attained a BP <140/90 mmHg, whereas only 10.2% (5/49) patients with diabetes and/or CKD attained the target value of <130/80 mmHg (< 0.001). In the first group, 45.5% (71/156) patients had stage 1 hypertension and 9% (14/156) had stage 2 hypertension, according to the JNC 7 classification of BP for adults. In the second group, 31% (15/49) had their BP within 120-139/80-89, 43% (21/49) had stage 1 hypertension and 16% (8/49) had stage 2 hypertension. Overall, only 37% (76/205) of hypertensive patients had their BP controlled according to the JCN 7 guidelines. All 5 patients that achieved BP control in the second group were males, although a significant difference between sexes was not achieved (*P *= 0.050). Likewise, the proportion of males in the total of patients that achieved BP control in the first group (39%, 28/71) was not significantly different from the proportion of males in the total of patients that did not achieve BP control (35%, 30/85, *P *= 0.594). When considering obese hypertensive patients (body mass index ≥30), 36.4% (12/33) achieved BP control in the first group, which is arithmetically lower, although not significantly (*P *= 0.232), than the BP control obtained in nonobese patients (48.0%, 59/123). In the diabetes and CKD group there was also not a statistically significant difference (P = 1) in the BP control between obese (11.8%, 2/17) and nonobese patients (9.4%, 3/32); however, the number of obese hypertensive patients in this group was too small to extract accurate statistical conclusions.

Among patients initiating hypertension consultation within the study period, 32.1% (9/28) had stage 2 hypertension in the first appointment, but this figure decreased to 3.6% (1/28) in the last consultation (*P *= 0.012), and was not significantly different from the remainder overall population at the last appointment (11.9%, 21/177, *P *= 0.322). However, there was a significant higher percentage of patients with stage 2 hypertension in the first medical appointment at the hypertension/dyslipidemia clinic, when compared to the total study population at the last appointment (*P *= 0.009). Despite this improvement in BP measures in patients initiating hypertension consultation within the study period, there was not a statistically significant (*P *= 0.188) improvement in the percentage of patients with controlled BP, according to JNC 7 guidelines, between the first and the last appointments. However, in these patients, there was a significant decrease in the mean ± SD systolic BP from the first to the last appointment (149.4 ± 17.1 versus 138.1 ± 11.0, *P *< 0.001). Conversely, the decrease reported in the diastolic BP from the first to the last appointment was not statistically significant (87.3 ± 13.2 versus 83.7 ± 9.3, *P *= 0.072). The average interval time between the first and the last appointment was 6 months in this subgroup of patients, with an average of 4 medical appointments per patient.

The attainment of BP control per age-group and cardiovascular risk are represented in Table 2. Concerning the lower cardiovascular risk group, the percentage of patients achieving BP control was not significantly different (*P *= 1) in the age groups ≥18 - <35 years (50%) and 35-64 years (54%), but was significantly lower (*P *= 0.001) in the age group ≥65 years (25%). When considering the higher cardiovascular risk group, the percentage of patients achieving BP control was only 10% for both age groups involved.

### Antihypertensive therapy

In all, 196 patients were prescribed with antihypertensive medication. The remaining 9 patients have only been counselled to adopt lifestyle modifications in order to control their BP, and 6 of these were already controlled without any antihypertensive medication. It is worth noting that these 9 patients were significantly younger than the average population, with an overall mean age of 44 ± 13 years (P < 0.001). The recommended lifestyle changes for BP control were in accordance with the JNC 7 guidelines and included: (1) weight loss in the overweight patients, (2) reduced sodium intake, (3) increased physical activity, and (4) limited alcohol consumption.

Overall, the patients took a mean ± SD of 2.7 ± 1.4 antihypertensives daily. Thiazide-type diuretics were the most frequently prescribed antihypertensive agents, with a total of 66.8% (137/205) patients being prescribed with this class of drugs, followed by angiotensin receptor blockers (ARBs) (60.1%, 123/205) and beta-blockers (42.6%, 87/205). These three classes of antihypertensives were the most prescribed (and in the same ranking order) both in patients without diabetes and/or CKD and in patients with these diseases (Table 4). However, the first group took 2.5 ± 1.3 antihypertensives daily, whereas in the second group this figure was 3.2 ± 1.3 (*P *= 0.005).

**Table 4:** Antihypertensive medication prescribed to hypertensive patients

Antihypertensive Drug	All patients (n = 205) (%)	Patients with target BP <140/90 mmHg (n = 156) (%)	Patients with target BP <130/80 mmHg (n = 49) (%)	*P *Value*
**Loop diuretics**	**13.2**	**11.5**	**18.4**	0.218
Furosemide	13.2	11.5	18.4	

**Thiazide diuretics**	**66.8**	**64.1**	**75.5**	0.139
Altizide	1.0	0.6	2.0	
Hydrochlorothiazide	50.7	46.8	63.3	
Indapamide	15.1	16.7	10.2	

**Potassium-sparing diuretics**	**3.9**	**1.3**	**12.3**	0.003
Spironolactone	2.9	1.3	8.2	
Triamterene	1.0	0.0	4.1	

**Renin inhibitor**	**2.0**	**1.9**	**2.0**	1
Aliskiren	2.0	1.9	2.0	

**Angiotensin-converting enzyme inhibitors**	**29.8**	**27.5**	**36.8**	0.221
Cilazapril	1.0	1.3	0.0	
Enalapril	11.7	7.7	24.5	
Imidapril	0.5	0.6	0.0	
Lisinopril	3.4	3.2	4.1	
Perindopril	6.8	9.0	0.0	
Ramipril	5.9	5.1	8.2	
Trandolapril	0.5	0.6	0.0	

**Angiotensin II-receptor antagonists**	**60.1**	**58.4**	**65.3**	0.383
Candesartan	6.3	7.7	2.0	
Eprosartan	2.0	1.3	4.1	
Irbesartan	8.8	8.3	10.2	
Losartan	12.2	13.5	8.2	
Olmesartan	2.0	2.6	0.0	
Telmisartan	15.1	13.5	20.4	
Valsartan	13.7	11.5	20.4	

**Calcium channel blockers**	**39.7**	**36.4**	**48.9**	0.121
Amlodipine	21.5	18.6	30.6	
Felodipine	2.0	1.9	2.0	
Lercanidipine	3.9	3.8	4.1	
Nifedipine	5.4	5.1	6.1	
Nimodipine	0.5	0.6	0.0	
Nitrendipine	1.0	1.3	0.0	
Diltiazem	5.4	5.1	6.1	

**Beta-blockers**	**42.6**	**41.6**	**45.0**	0.689
Atenolol	5.9	6.4	4.1	
Bisoprolol	5.9	6.4	4.1	
Metoprolol	0.5	0.6	0.0	
Nebivolol	23.9	21.2	32.7	
Propranolol	0.5	0.6	0.0	
Carvedilol	5.9	6.4	4.1	

**Central alpha-2 agonists**	**10.8**	**10.8**	**10.2**	0.888
Clonidine	0.5	0.6	0.0	
Methyldopa	0.5	0.6	0.0	
Rilmenidine	9.8	9.6	10.2	

*P values are for comparison between the two last columns.

**Figure 1:**
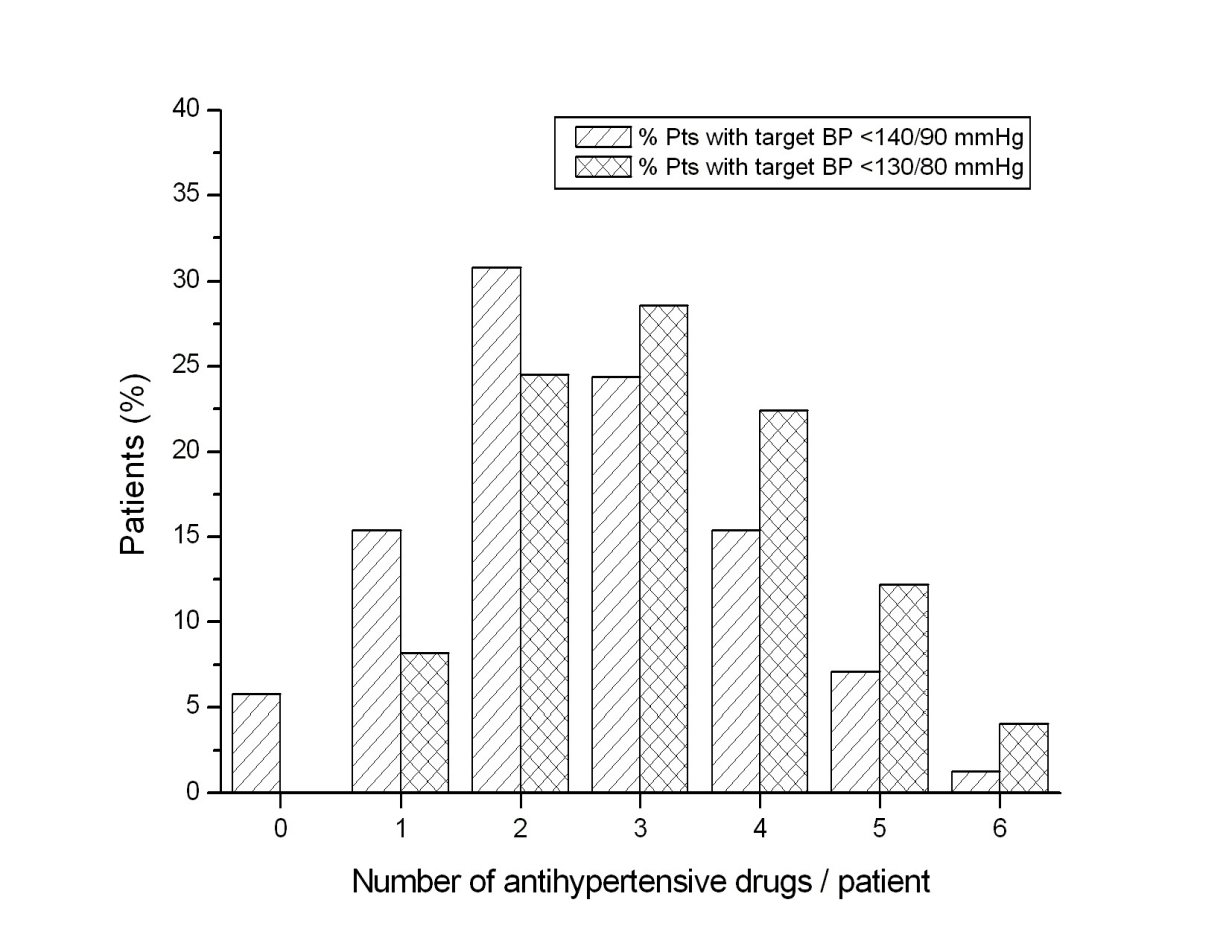
**Number of antihypertensive medications per patient**.

The number of antihypertensive drugs taken per patient ranged from 1 to 6 (Figure 1). The percentage of patients taking 1 or 2 antihypertensive drugs were higher in hypertensives with a target BP of <140/90 mmHg; on the contrary, the percentage of patients taking 3, 4, 5 or 6 antihypertensive drugs were higher in patients with a lower target BP (<130/80 mmHg).

**Figure 2:**
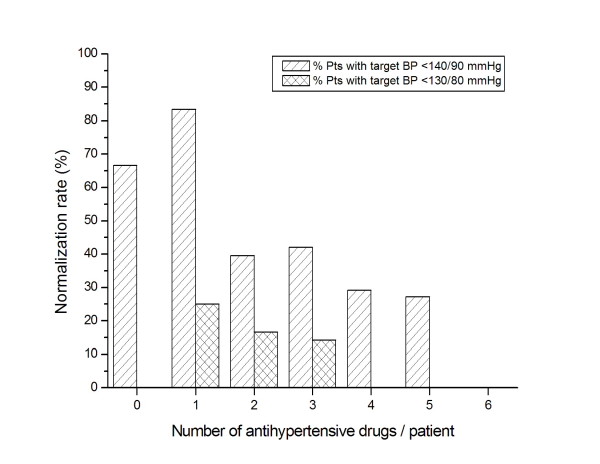
**Blood pressure normalization according to the number of antihypertensive drugs**.

As shown in Figure 2, the rate of BP normalization seemed to be inversely related to the intensity of the treatment: in patients prescribed with antihypertensives, the higher the number of drugs, the lower the rate of BP normalization, with a significant correlation between the two variables. In patients with target values of <140/90 mmHg the correlation between those variables was -0.916 (*P *= 0.010), and in patients with target values of <130/80 mmHg the correlation was -0.935 (*P *= 0.006). It should be noted that in the first group of patients BP control was not achieved only with a 6-drug combination regimen therapy, whereas in the second group it was not achieved with 4-, 5- or 6-drug combinations (Figures 1 and 2). Resistant hypertension must be strongly suspected at least in 4 patients in whom BP control was not achieved even with a 6-drug combination therapy.

For patients without compelling indications, beta-blockers were the most frequently prescribed antihypertensive agents as monotherapy; for those with compelling indications angiotensin-converting enzyme inhibitors (ACEIs) were the most frequently prescribed. In all, the most common 2-drug combinations were thiazide diuretic plus ARB (37% of all patients receiving 2 drugs) and thiazide diuretic plus ACEI (22% of all patients receiving 2 drugs). These 2-drug combinations were the most common both in patients without or with compelling indications.

Almost all patients with comorbid diabetes (95.9%) were treated with an ACEI or ARB, according to the best practice recommendations; also, according to best practice, the two reported hypertensive patients (100%) with comorbid congestive heart failure were treated with ACEI, ARB, or beta-blocker; however, only one patient (25%) with a history of myocardial infarction was treated with beta-blockers.

## Discussion

The results presented in this study describe the demographic and clinical characteristics of hypertensive patients attending the medical consultation of hypertension/dyslipidemia in a university teaching hospital located in the Eastern Central Region of Portugal for routine follow-up, focusing on the level of hypertension control and antihypertensive therapy.

According to a survey conducted in 2003 [2], of the total number of hypertensives in the Central Region of Portugal who reported taking their medication regularly, only 26.1% had their BP measurements <140/90 mmHg, which is significantly smaller than the 44.4% obtained in our study (Table 3). This difference possibly points to an improved current care of the hypertensives included in our study when compared to those included in the above mentioned survey. Increased awareness of hypertension and the importance of lower BP may have prompted Portuguese providers and patients to more aggressively treat high BP, especially after the publication of the JNC 7 report in 2003. Till now there has been no data about the percentage of treated hypertensives in clinical practice, in this Portuguese region, with their BP controlled according to the JNC 7 guidelines. Our study revealed that 37.1% of hypertensive patients had their BP controlled according to those guidelines, with the percentage of patients without diabetes or CKD attaining BP control (45.5%) significantly higher (*P *< 0.001) than the percentage of hypertensive patients with diabetes or CKD (10.2%). It should be noted that the reported levels of BP control can vary greatly depending on the study population, methods and time frame [4,5]. For example, in one study based on data from the US National Health and Nutrition Examination Survey 2003-2004, the BP control rate (to <140/90 mmHg) was 56.6% in treated hypertensives, and 37.5% in treated hypertensive persons with diabetes mellitus (for whom the goal BP is <130/80 mmHg) [6]. In a regional survey performed in the middle-West of France and involving 1050 treated hypertensives, Ragot *et al. *reported that 39% of patients had BP figures <140/90 mmHg and only 13% of the diabetic population were normalized according to the international recommendations (<130/80 mmHg) [7]. In a more recent retrospective observational study conducted in the United States, Jackson *et al. *[5] reported a BP control of 49.3% in an after-JNC 7 cohort. In this cohort, a significantly higher percentage of nondiabetic patients achieved BP control compared with those with comorbid diabetes (60.9% versus 29.4%). Similarly, Andros *et al. *[8] conducted a retrospective observational study of BP control in an insured diabetic population, obtaining a BP control rate (defined by JNC 7) of 28%, similar to the 29.4% obtained by Jackson *et al. *[5]. The results obtained in our study are less optimistic, especially when considering the percentage of BP control attained by hypertensives with diabetes or CKD (10.2%) and seem to be similar to those obtained by Ragot *et al*. in a French population [7]. The percentage of patients taking a higher number of antihypertensive drugs were higher in patients with a lower target BP (<130/80 mmHg) (Figure 1), suggesting that an effort is being made to further lower BP in this hypertensive subgroup. However, our results insinuate that prescribers may not be fully following the JNC 7 recommended BP targets, especially those related to hypertensives with diabetes or CKD, because the above mentioned studies demonstrated that it is possible to obtain a higher BP control in this hypertensive subpopulation. In fact, there was no significantly difference in the percentage of hypertensives in each cohort that achieved a BP of <140/90 mmHg (45.5% versus 40.8%; *P *= 0.564). Thus, our findings indicate that patients who would benefit most from tighter control of BP, especially those with compelling indications, appeared to do worse than those with uncomplicated hypertension. Furthermore, it seems to us that stage 1 hypertension is not seen as a major problem because a rather significant percentage of hypertensive patients is maintained in this hypertension stage (Table 3). Other possible underlying causes of poor BP control are guidelines unawareness and therapeutic inertia on the part of providers and poor adherence and persistence with prescribed medications and lifestyle modifications by patients. Results of studies suggest that antihypertensive medications are frequently not intensified when BP remains uncontrolled, termed clinical inertia [9-11]. In the recent Harris Survey [12], more than 30% of hypertensive patients reported that their medication was not changed or increased despite the fact that their BP was still >140/90 mmHg. Antihypertensive medication nonadherence is another major factor that must be thought about when considering the possible reasons for the inadequate BP control. Indeed, there is a large proportion of patients in our study (65%) who had hypertension for over five years. It is known that patient persistence with prescribed therapy for any chronic disease typically declines over time, and hypertension is no exception [13-15].

Of particular note is that the BP control rate in patients with target values of <140/90 mmHg was significantly lower in older hypertensive individuals (25%; Table 2), which is in accordance with rates mentioned in the literature and are largely due to poor control of systolic BP [3,16]. Obesity is identified as one cause of resistant hypertension [17] and there was a trend, albeit not significant, toward higher BP control between nonobese hypertensive patients (40% vs 28%, 0.127). The nonsignificant difference in BP control rate between obese and nonobese in our analysis could be because of the limited sample size, because >75% of the patients with diagnosed arterial hypertension were nonobese (body mass index <30).

The differences in rates of BP control between males and females were not significant in the <140/90 mmHg and in the <130/80 mmHg BP targeted population. Our results are in accordance with a recent study assessing gender difference in BP control that used the same cut points of uncontrolled BP defined by the JNC 7 [18].

Our results also indicate that there is a significantly higher percentage of patients with stage 2 hypertension in the first medical appointment at the hypertension/dyslipidemia clinic, when compared to the same patients and to the total study population at the last medical appointment. The decrease in stage 2 hypertension in patients attending the hypertension/dyslipidemia clinic for the first time was paralleled by a significant decrease in the systolic BP from the first to the last appointment (from 149.4 ± 17.1 to 138.1 ± 11.0, *P *< 0.001). These facts suggest that clinically important BP decreases can be achieved soon after hypertension medical appointment initiation.

Results from this study indicate that prescribers are following the JNC 7 drug therapy recommendations, including the use of thiazide-type diuretics as preferred initial agent in patients without compelling indications and those related to compelling indications. In fact, the use of ACEIs and ARBs in patients with diabetes and/or congestive heart failure coincides with JNC 7 recommendations. Data reported here do not, however, suggest that postmyocardial infarction patients are being mostly treated with beta-blockers.

The observed relationship between increased number of antihypertensive drugs and poorer BP control (Figure 2) could be explained by the fact that patients whose BP is more difficult to control are likely to be treated with multiple drugs. Thus, this measurement may be a consequence of poor BP control.

Several features of our study deserve further comment. First, the objective of this study was to describe levels of BP control in subgroups of hypertensive patients defined on the basis of important characteristics, and not to directly compare such levels across subgroups. For this motive, analyses adjusted for other characteristics were not conducted. Second, apart from patients initiating hypertension appointments within the study period, in which the BP measurements in the first appointment was also considered, BP control was determined based on the last available readings during the retrospective study period. These measurements may or may not be representative of the adequacy of control over the entire corresponding period. Third, drug information in the study database is confined to prescriptions written and not necessarily to those dispensed or used. Whether written prescriptions were filled by the patients and the level of medication adherence among patients who did fill the prescriptions is unknown. Finally, because of the retrospective design of the study, some data were not available or not able to be validated during the data collection process (Table 1). Although the missing data were quite reduced, the possibility exists that certain patient characteristics, conditions and risk factors were over- or under-represented.

## Conclusions

The findings of this study indicate that many treated hypertensive patients fail to achieve BP control according to the JNC 7 guidelines. The most troublesome result, however, is the extremely low BP control rate attained by hypertensives with diabetes or CKD, i.e., BP control is worse among patients who are at high risk for adverse outcomes and may benefit the most from lower BP levels. The results obtained in this hypertensive subpopulation are significantly worse than those obtained by other authors. Even the BP control in hypertensives without diabetes or CKD can be improved if we compare our results with those obtained in clinical practice in other countries. Additional research is needed to identify the underlying causes of poor BP control, such as guidelines unawareness and therapeutic inertia on the part of providers and poor adherence and persistence with prescribed medications and lifestyle modifications by patients, so that team-based health care providers and patient interventions can be established that may address these causes.

## Competing interests

The authors declare that they have no competing interests.

## Authors' contributions

MM participated in the acquisition, analysis and interpretation of data and has been involved in drafting the manuscript. SR participated in the analysis and interpretation of data and has been involved in drafting the manuscript. LP participated in the design of the study and performed the statistical analysis. MCB conceived the study, participated in its design and coordination, helped to draft the manuscript and gave final approval to the version to be published. All authors read and approved the final manuscript.

## Pre-publication history

The pre-publication history for this paper can be accessed here:

http://www.biomedcentral.com/1472-6963/10/349/prepub
